# Feasibility and validity of the EQ-5D-3L in the elderly Europeans: a secondary data analysis using SHARE(d) data

**DOI:** 10.1007/s11136-022-03158-3

**Published:** 2022-05-27

**Authors:** Ines Buchholz, Ole Marten, Mathieu F. Janssen

**Affiliations:** 1 EuroQol Group, Rotterdam, The Netherlands; 2 Department of Health Economics and Health Care Management, Bielefeld University, 33615 Bielefeld, Germany; 3 Department of Medical Psychology and Psychotherapy Erasmus MC, Erasmus University, PO Box 2040, 3000 CA Rotterdam, The Netherlands

**Keywords:** EQ-5D, Quality of life, European ageing population, Elderly, Psychometric properties

## Abstract

**Purpose:**

To determine feasibility and validity of the EQ-5D-3L in the elderly European population.

**Methods:**

Secondary data analysis based on the study of health, ageing, and retirement in Europe (SHARE) to determine the percentage of missing items for EQ-5D dimensions and EQ VAS, and to demonstrate convergent/divergent validity with measures included in the SHARE survey. Known-groups validity was tested using literature-based hypotheses. Correlation coefficients and Cohen’s f are reported.

**Results:**

Missing values were below 3% across all EQ-5D dimensions and gender strata, slightly increasing with age. Individuals’ responses to each EQ-5D dimension were related to their ratings of other measures in expected directions. The EQ VAS and all EQ-5D dimensions (except anxiety/depression) moderately to strongly correlated with physical [e.g. number of limitations in activities of daily living (ADL): *r* = 0.313–0.658] and generic measures [CASP (control, autonomy, self-realization, pleasure)-19 scale, self-perceived health, number of symptoms: *r* = 0.318–0.622], while anxiety/depression strongly correlated with the EURO-D scale (*r* = 0.527). Both EQ-5D dimensions and EQ VAS discriminated well between two [or more] groups known to differ [e.g. anxiety/depression discriminated well between persons classified as depressed/not depressed using the EURO-D scale, *f* = 0.51; self-care differentiated best between individuals without and with 1 + ADL limitations, *f* = 0.69]. Sociodemographic variables like gender, education, and partner in household were hardly associated with EQ VAS scores (*f* < 0.25).

**Conclusion:**

With item non-response of less than 3%, good discriminatory, and construct properties, the EQ-5D-3L showed to be a feasible and valid measure in the elderly Europeans.

## Background

A long life can bring opportunities for the persons themselves, their relatives, and the society as a whole, but the extent to which a person can make use of additional lifetime essentially depends on at least one factor: health [1].

We can measure, describe, and compare health and its various manifestations—for one person over time, between persons, patient groups, and populations. The individual’s own perception of health is usually captured using patient-reported outcome measures. The EQ-5D belongs to this group of instruments.

Introduced in 1990 by the EuroQol Group and available in more than 170 languages, the EQ-5D-3L (originally “EQ-5D”) is one of the most widely used preference-accompanied measures [2]. It consists of two components—a descriptive system formed by five items each capturing one of five dimensions (mobility, self-care, usual activities, pain/discomfort, anxiety/depression) via three possible response options expressed as the level of problems (level 1: no problems, level 2: some or moderate problems, level 3: extreme problems, unable to or confined to bed), and a vertical thermometer scale ranging from 0 (“worst imaginable health state”) to 100 (“best imaginable health state”), called EQ VAS. The instrument has an important role in describing and understanding population health within and across countries [3–6]. Although there is a large body of literature on EQ-5D in the general adult population [7, 8], knowledge about the instrument’s measurement properties in the ageing population is scarce [6, 9, 10].

In the face of demographic change, the older and so-called “oldest-old” represent the broadest and fastest growing population groups. This development is no longer limited to the industrialized world and accompanied by a parallel epidemiological trend: the increase in chronic and degenerative diseases [11]. The WHO even speaks of “a dramatically increasing pace of population ageing around the world,” with lower- and mid-income countries now facing the greatest changes with respect to the shift in the ageing pyramid [1].

However, although additional lifetime can be spent in good health, it is evident that physical health problems occur more frequently in the elderly than in the overall adult general population and are increasing with age [12, 13]. A projection to 2060 by Sleeman et al. shows that the burden of serious health-related suffering will almost double by 2060, with the fastest increases among older people, and those with dementia [14].

Against this background, this contribution aims to extend our knowledge of the EQ-5D in the ageing population by investigating its feasibility and validity across 15 European countries.

## Methods

This secondary data analysis is based on the 4^th^ Wave data from the Survey of Health, Ageing and Retirement in Europe (SHARE) [15–18], a representative survey covering the elderly population (50+) in 15 European countries: Austria, Belgium, the Czech Republic, Denmark, France, Germany, Hungary, Italy, the Netherlands, Poland, Portugal, Slovenia, Spain, Sweden, and Switzerland. Data collection took place in 2011 (Germany/Poland: 2011/2012) and was based on computer-assisted personal interviewing (CAPI). Data of the three-level version of the EQ-5D were collected using a standardized short self-completion paper-and-pencil questionnaire (so-called “drop-off” questionnaire) [19, 20]. The questionnaire was always self-completed by the individual; proxy responses were not allowed. It begins with the five items of the descriptive system of the EQ-5D, followed by the EQ VAS, questions about payment for various types of care, out-of-pocket expenses, and four items about loneliness. For some countries, country-specific questions follow. The questionnaire could be given back to the interviewer or posted back in a provided envelope. There is no information on whether assistance is provided when completing the questionnaire in the presence of the interviewee. It should be noted that the instructions of the EQ-5D differ from the original.

Data analysis was done using STATA/SE 16.1 using all available EQ-5D data (no listwise deletion) of panellists aged 50 years and older. For analysis, we built age groups in five-year increments. A description of the data resource and the methodology can be found elsewhere [15, 17].

## Variables

For proving both construct validity and known-groups validity, a selection of variables and measures included in the SHARE survey was used depending on the type of validity and the underlying construct. In the following, we are giving some information on the operationalization and definition of those variables. Detailed information (inclusive references) can be found elsewhere [21].

Sample characterization is based on the following variables from SHARE Wave 4: age, gender, country, education [years in school and International Standard Classification of Education (ISCED)], marital status, mean EQ VAS, current job situation, self-perceived health, number of symptoms for at least six months, number of limitations in activities of daily living (ADL), body mass index (BMI), and current smoking behaviour.

### Physical measures

The number of limitations in six everyday self-care activities such as walking, eating, and toileting is expressed in a 0–6 ranging *ADL* index with higher values representing more limitation in activities which are fundamental for maintaining independence [22].

The *IADL* index describes the number of limitations with seven instrumental activities of daily living like taking medications, making telephone calls, and managing money. The score ranges from 0 to 7 with higher values expressing more limitations in IADL [22].

Long-standing activity limitations (6 month or more) are measured with a global single-item indictor which was developed for comparing health expectancy and disability across Europe. This dichotomous (0 = limited, 1 = not limited) global activities limitation index (*GALI*) refers to general health problems and activities people usually do [23].

*Physical inactivity* is operationalized as a dichotomous measure that comprises the answers of two items “We would like to know about the type and amount of physical activity you do in your daily life. How often do you engage in *vigorous* physical activity, such as sports, heavy housework, or a job that involves physical labour?”, and “How often do you engage in activities that require a *moderate* level of energy such as gardening, cleaning the car, or doing a walk?” with “1” representing “never vigorous nor moderate physical activity”.

### Mental measures

The *EURO-D* scale used in SHARE comprises 12 items (e.g. depression, guilt, sleep, interest, appetite) that present common symptoms of late-life depression. The composition index ranges from 0 (not depressed) to 12 (very depressed) with values of 4 or higher indicating a case of depression (cut-off) [24].

*Anxiety* is operationalized as a 5-item indicator. Items such as “fear of the worst happening” or “fear of dying” stem from the Beck Anxiety Inventory and have four response options (1 = “never”, 2 = “hardly ever”, 3 = “some of the time”, 4 = “most of the time”). Higher values indicate higher levels of anxiety.

### Cognitive measures

The 10-words-recall test is used to measure cognitive impairments and dementia. Two scores are provided: the number of words the respondent is able to *immediately* recall after listening to a list of 10 words and the number of words the respondent is able to recall after a *delay time* (delayed recall) [25].

Another indicator of cognitive impairments, executive functions, is operationalized by a one item *verbal fluency* test. Respondents have 60 s to name as many different animals as they can think of. The score ranges from 0 to 100 and expresses the number of animals named within one minute. Animals were chosen because they represent a clear and popular semantic category across languages and cultures.

*Memory* performance is measured using a single item (“How would you rate your memory at the present time? Would you say it is excellent, very good, good, fair or poor?”) with a score ranging from 1 (“excellent”) to 5 (“poor”).

The *mathematical performance* is measured by five items that test subtraction calculation skills, e.g. “Now let's try some subtraction of numbers. One hundred minus 7 equals what?”, “And 7 from that?”. The score ranges from 1 (“bad”) to 5 (good).

*Temporal orientation* is operationalized as the respondent’s orientation to date, month, year, and day of week measured by four items (e.g. “Which month is it?”). The score ranges from 0 to 4 with higher values representing better orientation.

### General health and other measures

The *BMI* was calculated according to the WHO definition of 1995 and expresses the weight (in kg) divided by the square of the height (metres^2^).

*Self-perceived health status* was assessed using two measures: a single item of self-perceived health with response categories based on the SF-36 (ranging between “excellent” and “poor”) and a 12-item revised version of the CASP-19 scale (the name is an acronym of the four subscales: control, autonomy, self-realization, pleasure). Since for each item, there are 4 response options, the CASP index ranges from 12 (low quality of life) to 48 (high quality of life) [26, 27].

A maximum value of the *grip strength* measurements of both hands was generated for respondents with two valid measures for each hand and if the two measures for one hand do not differ more than 20 kg.

*Number of symptoms* reflects the number of health conditions the respondent has been bothered for at least the past six month. For assessing this, respondents were asked to look at a card with several health conditions of which they can choose such as pain in the back, knees, hips, or any other joints, sleeping problems or incontinence.

## Feasibility

As a starting point of our analysis, information about the nature, the extent, and the selectivity of missing data and the (assumed) underlying mechanisms was gained by frequency count, and analysis of missing patterns [28]. If it could be ruled out that data are missing not at random (e.g. individuals with a history of anxiety disorder are more likely to omit the EQ-5D item anxiety/depression), feasibility was defined as the unweighted percentage of “not answered” items. For this purpose, we tested the relationship between the frequency distributions of two variables (e.g. using cross-tabulations and *χ*^2^-tests) and checked missing correlation patterns (results not shown). Instead of a completion rate, we are reporting the percentage of not answered items for each and all EQ-5D dimensions, for the EQ VAS, and for both EQ-5D dimensions and EQ VAS by gender, age groups, and country. The data were not weighted as it more directly reflects the feasibility of the survey.

## Validity

*Construct validity* was examined by correlating EQ-5D dimensions and EQ VAS with other measures supposed to measure a similar (convergent validity, e.g. measures of mental health like EURO-D scale and EQ-5D anxiety/depression; CASP and EQ VAS) [21], or a dissimilar construct (divergent validity) [29–31]. Depending on the measurement level of the variables, the extent of relatedness of EQ-5D and other measures is expressed as a Pearson correlation coefficient (interval scale) or Spearman’s rho (at least one variable ordinal; both range: − 1 to + 1) and interpreted as follows: poor (*r* < 0.3), moderate (*r* ≥ 0.3 to *r* < 0.5), large (*r* ≥ 0.5) [32]; only correlations significant at the 1% level after Bonferroni adjustment are reported.

*Known-groups validity* was determined by comparing groups known to differ based on e.g. sociodemographic and health variables like age, gender, marital status, number of symptoms, and self-reported health. Results are expressed as anchor-based distribution of responses (e.g. percentage of “no problems” by category of each variable, mean EQ VAS values by category of each variable). For comparison reasons, we are reporting Cohen’s f as an effect size measure. *F* estimates the proportion of variance explained by the categorical (grouping) variable [30, 33]. According to Cohen, *f* is interpreted as follows when used in an analysis of variance (ANOVA):

- *f* = 0.10 small effect
- *f* = 0.25 medium effect
- *f* = 0.40 large effect

For two group comparisons (t test, e.g. comparison of mean EQ VAS values between men and women), we derived Cohen’s f from Cohen’s d by dividing d by 2; for more than two group comparisons (e.g. comparison of mean EQ VAS values across age groups), we converted eta squared (from ANOVA) into Cohen’s f. To examine the association between two categorical variables (e.g. EQ-5D dimension mobility and BMI), we calculated Cramér's ν. V takes values between 0 and 1, with higher values indicating a stronger relationship. In the literature, ν is interpreted differently; we interpret *ν* = 0.1 as a small, *ν* = 0.3 as a medium, and *ν* = 0.5 as a large effect.

The complete validity analysis is based on weighted data. Whenever useful, results are reported stratified by country, age group, and/or gender.

## Results

Data from more than fifty thousand panellists were included, with a mean age of 66 years, of which 56% were female. The division into age groups in five-year steps resulted in a nearly equivalent distribution with 10–19% of panellists in each age group. One in four panellists was in school for at least 12 years; two thirds were married or living in a registered partnership, and 58% were retired. Although only less than one fourth of the panellists reported no symptoms, 88% stated to have no limitations with ADL, and more than 40% reported fair or poor self-rated health; mean EQ VAS was 71. Panellist’s characteristics are presented in Table 1.

**Table 1:** Sample characteristics

Sample size (n)	50,013
Female panellists (%)	55.6
Age in yrs, mean ± SD (range)	65.9 ± 10.0 (50–111)
*Age groups (%)*	
50–54 yrs	13.4
55–59 yrs	17.7
60–64 yrs	18.9
65–69 yrs	15.8
70–74 yrs	13.1
75–79 yrs	9.9
80+ yrs	11.3
*Education (%)*	
≥ 12 yrs of schooling	25.6
*Marital status (%)*	
Single/divorced/separated	16.3
Married/registered partnership	68.3
Widowed	15.5
*Current job situation (%)*	
Retired	57.9
Employed or self-employed	25.1
Unemployed	3.1
Permanently sick or disabled	3.5
Homemaker	9.1
Other	1.2
*Country shares in the sample, n (%)*	
Austria	5088 (10.2)
Belgium	5175 (10.4)
Denmark	2226 (4.5)
France	5635 (11.3)
Germany	1608 (3.2)
Hungary	2993 (6.0)
Italy	3505 (7.0)
Poland	1721 (3.4)
Portugal	1955 (3.9)
Slovenia	2702 (5.4)
Spain	3647 (7.3)
Sweden	1963 (3.9)
Switzerland	3668 (7.3)
The Czech Republic	5379 (10.8)
The Netherlands	2748 (5.5)
*EQ-5D (%)*	
*Mobility*	
No problems walking	32,885 (72.0)
Some problems walking	11,607 (25.4)
Confined to bed	336 (0.7)
Not answered	856 (1.9)
*Self-care*	
No problems with self-care	40,712 (89.1)
Some problems washing or dressing	3275 (7.02)
Unable to wash or dress	670 (1.5)
Not answered	1028 (2.3)
*Usual activities*	
No problems with usual activities	35,721 (78.2)
Some problems with usual activities	7870 (17.2)
Unable to perform usual activities	1151 (2.5)
Not answered	942 (2.1)
*Pain/discomfort*	
No pain or discomfort	21,377 (46.8)
Moderate pain or discomfort	20,920 (45.8)
Extreme pain or discomfort	2521 (5.5)
Not answered	875 (1.9)
*Anxiety/depression*	
Not anxious or depressed	33,545 (73.4)
Moderately anxious or depressed	10,040 (22.0)
Extremely anxious or depressed	1111 (2.4)
Not answered	995 (2.2)
EQ VAS, mean (SD)	71.2 (19.7)
*Self-perceived health US version (%)*^a^	
Excellent	7.3
Very good	17.6
Good	36.3
Fair	27.1
Poor	11.7
*Number of symptoms (%) for the past 6 month at least*	
0	24.4
1	28.2
2	17.3
3 +	30.0
*Number of limitations with activities of daily living (ADL) (%)*	
0	88.1
1	6.1
2 +	5.8
*Body mass index (BMI) (%)*	
< 18.5 (underweight)	1.3
18.5–24.9 (normal)	35.0
25–29.9 (overweight)	39.9
30+ (obese)	20.1
*Current smoking (%)*	
Yes, currently smoke	19.1
Never smoked daily for at least one year	54.6
No, I have stopped	26.3

Unweighted data

*yrs* years

^a^ “Would you say your health is… excellent/very good/good/fair/poor?”

## Feasibility

**Fig. 1:**
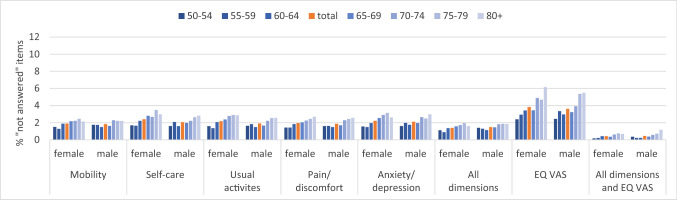
Percentage of not answered items by gender and age group. Unweighted data

In the total SHARE sample, only 3% of all respondents have at least one missing value on EQ-5D; responses on the descriptive system are completely missing in 1.32%, on the whole instrument in 0.4%, and on the EQ VAS in 2.8% of the panellists. After stratifying by age and gender, missing values are generally below 3% across all dimensions and strata but most frequently missing in the dimensions self-care (women: 2.4%, men: 2.1%) and anxiety/depression (women: 2.2%, men: 2.1%) (Fig. 1). Overall, there is an increasing trend with higher age where missing responses peak in panellists aged 75–79 years. However, missing values are similarly distributed between men and women (Fig. 1); for EQ VAS, they range from 2.4 to 6.2%, and 2.4 to 5.5% in women and men, respectively, thus, generally higher than the rate of missing values on the descriptive system across age groups. Similarly, we observe a stronger increase in item non-response with advancing age resulting in the highest proportion of unanswered EQ VASs in the oldest-old panellists (Fig. 1).

**Fig. 2:**
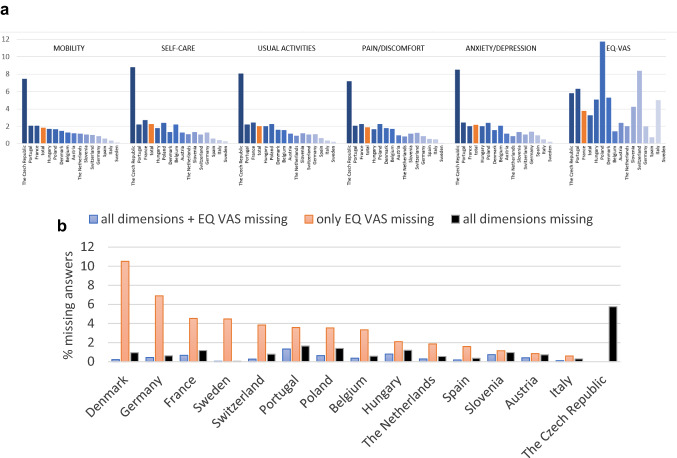
**a** Percentage of not answered items by country. Unweighted data. **b** Percentage of not answered items by country: all dimensions, only EQ VAS, and complete EQ-5D (dimensions and EQ VAS) missing; countries sorted decreasing by “only EQ VAS missing”. Unweighted data

Considering the total sample, except the Czech Republic, the proportion of not answered EQ-5D items is well below 2% across the five dimensions (Fig. 2a). However, there is great variability at the country level. Responses to the five dimensions are most frequently missing in the Czech Republic ranging between 7.2 and 8.5%, whereas Spain, Italy, and Sweden have consistently less than 1% missing values in all dimensions (Fig. 2a). Again, regarding the EQ VAS, missing values are slightly more prevalent (3.8%). Denmark is notable here with 10.8% missing EQ VAS responses, while items on the descriptive system are missing for less than 1.8% of the panellists indicating some discrepancy between the two components of the EQ-5D (Fig. 2b). This contrasts with the Czech Republic, with the highest number of missing values on the descriptive system but no missing answers on the EQ VAS. A similar but less pronounced gap can also be observed for Germany (EQ VAS: 8.4%, dimensions: 0.9–1.4%) and Sweden (EQ VAS: 5.0%, dimensions: 0.06–0.3%). On the contrary, Slovenia and Italy have consistently few missing responses (for both EQ-5D dimensions and EQ VAS).

## Construct validity

**Fig. 3:**
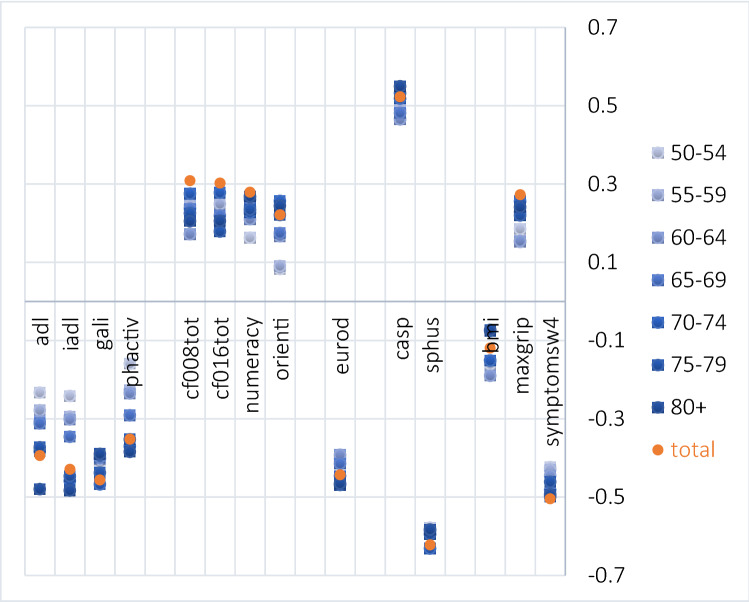
Construct validity—correlations between EQ VAS and physical, cognitive, mental, quality of life, and general health measures by age group; weighted by [aweight = cciw_w4]. Variable names correspond to the labels in the SHARE manual “Scales and Multi-Item Indicators” [21], codebooks and datasets: adl activities of daily living (0–6); iadl instrumental activities of daily living (0–7); gali global activity limitation index (0/1); phactiv physical inactivity (0/1); cf008tot 10-words-recall test (immediately recall after listening to a list of 10 words, 0–10); cf016tot delayed recall test (0–10); numeracy mathematical performance (0.5); orienti temporal orientation (0–4); eurod-modified EuroD, measure of late-life depression (0–12); casp 12-item (modified) version of the CASP-19, measure of quality of life (0–12); *sphus* self-perceived health US version (1–5), *bmi* body mass index, *maxgrip* maximum grip strength, *symptomsw4* number of symptoms

Except for anxiety/depression, all EQ-5D dimensions moderately to strongly correlate with most physical measures, while anxiety/depression strongly correlates with the EURO-D (*r* = 0.527), demonstrating satisfactory convergent and divergent validity (Table 2). Moreover, all EQ-5D dimensions at least moderately and the EQ VAS strongly correlate with generic measures of self-perceived health status, namely CASP (*r* = 0.323–0.523), self-perceived health (*r* = 0.316–0.622), and number of symptoms (*r* = 0.352–0.520). For divergent measures (BMI, maximum grip strength, and all cognitive measures), correlations with EQ-5D dimensions or EQ VAS are small ranging between 0 (e.g. self-care and nervous) and 0.308 (e.g. EQ VAS and immediate 10-words-recall test); for convergent measures, correlations are predominantly found in the range between 0.35 and 0.65, with the highest correlation of *r* = 0.658 between usual activities and number of limitations in ADL, and *r* = 0.622 between self-perceived health and EQ VAS (Table 2). For physical and cognitive measures, the correlations with the EQ VAS are increasing with age, while the correlation with self-perceived health is stable across age groups (*r*_50-54yrs_ = − 0.578 to *r*_70-74yrs_ = − 0.632, Fig. 3). This is particularly pronounced for ADL (*r*_80+yrs_ = − 0.480, *r*_50-54yrs_ = − 0.233) and IADL (*r*_80+yrs_ = − 0.483, *r*_50-54yrs_ =  − 0.242) with twice as high correlation coefficients in the group of the oldest compared to the youngest panellists.

**Table 2:** Construct validity—correlation matrix; correlations between EQ-5D dimensions or EQ VAS and different measures (small < 0.3, *moderate ≥ 0.3* to < 0.5, **large ≥ 0.5**), depending on measurement level Pearson correlation coefficient or Spearman’s rho

Type of measure	Measures (range of score)^SHARE variable label^	Mobility	Self-care	Usual activities	Pain/discomfort	Anxiety/depression	EQ VAS
Physical measures	Number of limitations in adl (0–6)^adl^	*0.445*	**0.658**	**0.539**	*0.318*	0.235	− *0.394*
	Number of limitations in iadl (0–7)^iadl^	*0.440*	**0.599**	**0.564**	*0.313*	0.257	− *0.429*
	Global activity limitations index (0/1)^gali^	*0.452*	0.274	*0.415*	**0.500**	0.253	− *0.456*
	Physical inactivity (0/1)^phactiv^	*0.348*	*0.417*	*0.398*	0.240	0.204	− *0.352*
Mental measures	EuroD (SHARE version, 0–12)	*0.307*	0.272	*0.363*	*0.366*	**0.527**	− *0.443*
	Fear of the worst happening (1–4)^mh023_^	0.184	0.172	0.201	0.190	*0.300*	− 0.278
	Nervous (1–4)^mh024_^	0.120	0.000	0.163	0.188	0.345	− 0.245
	Hands trembling (1–4)^mh025_^	0.240	0.242	0.287	0.232	0.282	− *0.329*
	Fear of dying (1–4)^mh026_^	0.175	0.193	0.223	0.161	0.247	− 0.288
	Felt faint (1–4)^mh027_^	*0.322*	0.274	*0.346*	*0.332*	0.297	− *0.412*
Cognitive measures	10-words-recall test, immediate rec. (0–10)^cf008tot^	− 0.235	− 0.253	− 0.270	− 0.173	− 0.159	*0.308*
	10-words-recall test, delayed rec. (0–10)^cf016tot^	− 0.219	− 0.231	− 0.243	− 0.160	− 0.155	0.303
	Verbal fluency (0–100)^cf010_^	− 0.177	− 0.223	− 0.233	− 0.104	− 0.150	0.281
	Memory (1–5)^cf103_^	0.213	0.203	0.249	0.226	0.219	− 0.313
	Numeracy (1–5)^num^	− 0.203	− 0.210	− 0.230	− 0.171	− 0.174	0.279
	Temporal orientation (0–4)^orienti^	− 0.217	− 0.267	− 0.266	− 0.157	− 0.137	0.222
Self-perceived health status	Self-perceived health (1–5)^sphus^	*0.452*	*0.316*	*0.445*	*0.498*	*0.332*	− **0.622**
	CASP (12–48)	− *0.350*	− *0.323*	− *0.401*	− *0.332*	− *0.416*	**0.523**
General health measures	Body-mass-index^bmi^	0.152	0.057	0.083	0.128	0.023	− 0.119
	Max. grip strength^maxgrip^	− 0.235	− 0.206	− 0.270	− 0.234	− 0.214	0.273
	Number of symptoms^symptomsw4^	*0.446*	*0.352*	*0.462*	**0.520**	*0.379*	− **0.504**

Bold values indicate a large correlation coefficent

Italic values indicate a moderate

All correlation coefficients significant at the 1% level after Bonferroni adjustment. Data are weighted by the calibrated cross-sectional weights (Wave 4) provided for SHARE users (cciw_w4). For SHARE users and those who want to look up variables in manuals, variable names of the SHARE datasets are given in superscripts [19, 21]. For detailed information to all variables please see [21]

## Known-groups validity

The EQ-5D dimensions and the EQ VAS significantly and similarly differentiate among different stages of cognitive (e.g. memory, orientation), physical (e.g. ADL, IADL, GALI) and general health (e.g. number of symptoms, BMI, depression), demonstrating a satisfactory discriminative ability of the EQ-5D (Table 3). Regarding sociodemographic variables, we found that women rate their state of health worse than men, people living with a partner report fewer problems in all dimensions, and a social gradient regarding education and the current job situation. Again, the proportion of panellists reporting “no problems” and the mean EQ VAS are continuously decreasing with age; with the biggest declines for the oldest age group of 80+. EQ VAS scores show the largest relationship with general health measures like number of symptoms and self-perceived health with a Cohen’s *f* of > 0.8, followed by limitations with activities of daily living (ADL, IADL, GALI: *f* > 0.5) and a case of depression (which means a scale score of 4 or higher at the EURO-D scale, *f* > 0.4, Table 3). However, the association with sociodemographic variables like gender, education, and partner in household is small (*f* < 0.25), while correlations with cognitive measures, age, and current job situation are moderate (*f* > 0.3). The discriminatory ability of the individual EQ-5D dimensions is reflected in higher effect sizes in the respective corresponding groups, e.g. anxiety/depression separates best between individuals with and without depression (*f* = 0.51), self-care between individuals without ADL limitations and 1 + ADL limitations (*f* = 0.69), and pain/discomfort between individuals without IADL limitations and 1 + IADL limitations (*f* = 0.61).

**Table 3:** Known-groups validity, expressed as percentage of “no problems” by dimension and mean EQ VAS with Cohen’s f or Cramér’s ν as effect size measures

	Mobility	Self-care	Usual activities	Pain/discomfort	Anxiety/depression	EQ VAS mean [SE]
*Body mass index (in kg/m*^*2*^*)*						
Underweight (≤ 18.5)	64.1	83.4	63.4	35.5	62.4	63.7 [0.97]
Normal (18.6–24.9)	79.0	92.1	82.9	55.7	75.6	72.5 [0.16]
Overweight (25–29.9)	73.5	91.7	81.5	50.6	77.4	70.7 [0.14]
Obese (≥ 30)	59.2	87.4	71.7	37.9	70.9	65.3 [0.22]
	*ν* = 0.119	*ν* = 0.055	*ν* = 0.083	*ν* = 0.105	*ν* = 0.048	*f* = 0.139
*Number of symptoms*						
0	94.7	98.5	97.1	88.2	92.2	81.2 [0.14]
1	82.8	96.2	89.7	58.2	84.0	74.9 [0.15]
2	72.7	93.1	81.1	40.9	72.6	69.7 [0.20]
3	60.2	87.9	71.8	28.0	65.5	64.8 [0.27]
4	47.2	81.5	60.0	19.6	54.7	57.8 [0.37]
5	43.5	74.3	51.0	12.4	48.8	53.5 [0.45]
6	29.9	67.8	37.0	8.6	47.7	51.1 [0.54]
7	28.8	62.6	32.7	8.8	38.2	47.8 [0.84]
8	21.8	48.0	26.5	4.0	32.0	44.8 [0.84]
9	11.7	44.3	15.3	3.9	30.9	39.0 [1.50]
10	15.3	46.4	18.0	4.3	20.8	38.0 [1.93]
	*f* = 0.505	*f* = 0.388	*f* = 0.524	*f* = 0.648	*f* = 0.412	*f* = 0.844
*Self-perceived health*						
Excellent	97.0	99.1	98.6	89.1	94.0	89.1 [0.19]
Very good	94.8	98.6	97.1	80.4	89.7	83.5 [0.14]
Good	84.8	97.4	91.9	59.1	82.4	75.8 [0.11]
Fair	58.5	87.8	69.9	29.7	65.6	62.8 [0.15]
Poor	28.3	60.9	33.2	12.0	44.3	43.4 [0.29]
	*ν* = 0.348	*ν* = 0.288	*ν* = 0.368	*ν* = 0.399	*ν* = 0.258	*f* = 0.844
*Depression*^*eurodcat*^						
Yes (≥ 4)	54.4	80.6	61.3	29.1	45.4	59.2 [0.19]
No	80.5	95.2	87.9	58.8	87.7	74.9 [0.09]
	*f* = 0.278	*f* = 0.230	*f* = 0.324	*f* = 0.320	*f* = 0.507	*f* = 0.434
*Limitations with activities of daily living*^*adl2*^						
No limitations	79.5	96.6	86.6	55.1	78.0	72.9 [0.09]
1 + limitations	21.3	46.3	28.4	10.8	50.3	49.3 [0.32]
	*f* = 0.504	*f* = 0.668	*f* = 0.584	*f* = 0.380	*f* = 0.229	*f* = 0.644
*Limitations with instrumental activities of daily living*^*iadl2*^						
No limitations	80.9	96.6	88.5	56.3	87.7	73.8 [0.09]
1 + limitations	29.8	60.0	34.6	16.5	45.4	50.8 [0.25]
	*f* = 0.494	*f* = 0.516	*f* = 0.614	*f* = 0.384	*f* = 0.256	*f* = 0.639
*Limitations with activities*^*gali*^						
Not limited	92.2	98.6	96.2	73.6	85.1	78.7 [0.10]
limited	51.2	81.6	61.5	24.1	63.3	60.6 [0.14]
	*f* = 0.507	*f* = 0.285	*f* = 0.456	*f* = 0.577	*f* = 0.261	*f* = 0.514
*Temporal orientation*						
Bad	39.8	51.7	39.6	25.9	52.3	46.7 [1.58]
1	35.8	48.3	33.0	27.9	47.3	51.9 [2.27]
2	45.3	69.5	49.4	34.9	56.3	57.7 [1.21]
3	65.3	85.9	69.0	38.7	64.6	65.0 [0.42]
Good	79.4	93.0	83.2	56.8	76.1	72.8 [0.13]
	*ν* = 0.182	*ν* = 0.214	*ν* = 0.215	*ν* = 0.106	*ν* = 0.099	*f* = 0.227
*Memory (1–5)*						
Excellent	83.1	96.0	89.5	67.3	84.9	77.9 [0.32]
Very good	82.2	95.0	88.5	62.0	84.0	76.5 [0.18]
Good	75.6	93.8	84.0	51.5	78.3	72.0 [0.13]
Fair	64.9	87.1	71.5	39.7	65.6	64.7 [0.21]
Poor	38.5	61.0	41.6	21.0	45.4	49.5 [0.48]
	*ν* = 0.181	*ν* = 0.183	*ν* = 0.210	*ν* = 0.174	*ν* = 0.174	*f* = 0.362
*Numeracy (1–5)*						
Bad	53.1	72.0	55.2	37.2	59.0	56.3 [0.61]
2	69.9	86.5	73.5	45.4	67.3	65.8 [0.33]
3	78.0	93.1	82.1	55.7	73.8	72.3 [0.23]
4	81.8	95.3	85.8	57.7	78.8	75.4 [0.18]
Good	87.2	96.8	90.7	65.2	85.1	77.9 [0.25]
	*ν* = 0.168	*ν* = 0.186	*ν* = 0.190	*ν* = 0.130	*ν* = 0.124	*f* = 0.307
*Age (in yrs)*						
50–54	87.2	97.0	88.4	63.8	77.1	77.0 [0.23]
55–59	81.2	95.1	87.0	56.3	77.5	73.3 [0.21]
60–64	81.8	95.2	86.9	55.8	76.6	73.6 [0.19]
65–69	75.9	93.5	84.1	51.0	77.1	70.9 [0.23]
70–74	69.9	90.5	77.5	44.9	74.1	67.6 [0.27]
75–79	59.5	85.8	71.1	38.2	70.3	63.6 [0.32]
80 +	38.0	69.4	51.0	27.3	65.6	57.6 [0.32]
	*ν* = 0.211	*ν* = 0.183	*ν* = 0.190	*ν* = 0.141	*ν* = 0.060	*f* = 0.313
*Gender*						
Men	76.5	92.3	83.8	56.2	82.5	71.9 [0.13]
Women	68.9	88.8	75.7	44.1	67.9	68.3 [0.13]
	*f* = 0.084	*f* = 0.055	*f* = 0.097	*f* = 0.131	*f* = 0.167	*f* = 0.091
*Education*^*a*^						
< 12 yrs	70.9	88.3	75.3	48.7	71.3	68.1 [0.17]
≥ 12 yrs	86.0	95.6	88.5	62.6	78.7	76.4 [0.17]
	*f* = 0.173	*f* = 0.121	*f* = 0.159	*f* = 0.148	*f* = 0.087	*f* = 0.214
*Partner in household*						
Yes	76.7	92.9	83.1	53.2	77.7	71.9 [0.11]
No	63.5	85.3	71.9	42.3	68.2	65.9 [0.20]
	*f* = 0.140	*f* = 0.112	*f* = 0.127	*f* = 0.114	*f* = 0.105	*f* = 0.144
*ISCED-97*						
Code 1	64.9	83.7	70.8	43.7	64.9	63.6 [0.22]
Code 2	70.5	91.3	78.4	50.8	77.2	69.9 [0.23]
Code 3	74.7	93.5	83.1	49.9	77.6	71.7 [0.16]
Code 4	80.2	95.1	84.7	54.4	79.7	75.5 [0.46]
Code 5	81.2	95.9	88.3	58.6	82.9	77.0 [0.18]
Code 6	89.5	97.9	93.5	67.1	74.8	79.8 [0.78]
	*ν* = 0.124	*ν* = 0.122	*ν* = 0.135	*ν* = 0.107	*ν* = 0.106	*f* = 0.273
*Current job situation (%)*						
(self-)employed^b^	88.8	98.5	93.0	65.0	83.7	78.9 [0.14]
unemployed	81.2	97.7	87.8	50.6	72.5	70.8 [0.14]
other	67.9	84.8	77.0	50.8	63.9	66.4 [0.96]
homemaker	67.3	87.5	73.3	46.4	66.9	67.0 [0.31]
retired	66.8	88.2	76.1	44.4	74.0	67.4 [0.13]
perm. sick/disabled	48.2	74.4	48.3	25.9	49.5	53.4 [0.58]
	*ν* = 0.179	*ν* = 0.135	*ν* = 0.181	*ν* = 0.170	*ν* = 0.116	*f* = 0.326

Independence of the frequency distribution of two categorical and/or ordinal variables was assessed using *χ*^2^ tests with Cramér’s ν reported as an effect size measure

*eurodcat* Euro D scale, cut-off 4 (persons with a value of 4 and higher are classified as depressed), *ISCED* International Standard Classification of Education, *perm.* permanent

^a^Please note that the sample size of this variable is below 30,000, which is why we additionally report ISCED for which sample size is comparable to other variables in this dataset (missing values < 3%)

^b^Including working for family business

## Discussion

To the best of our knowledge, this is the first study to assess the feasibility, construct validity, and known-groups validity of the EQ-5D in a large-scale sample of older Europeans across 15 countries. Our findings indicate excellent feasibility of the EQ-5D descriptive system and the EQ VAS. Additionally, both showed a satisfactory construct validity and distinguished well between known-group differences. It is a major strength of this study that the overall analysis was conducted on the full-severity range using non-dichotomized EQ-5D data, which is common practice in the analysis of general population data.

Overall, just 3% of all respondents had one or more missing values, which implicates very good feasibility. The magnitude of missing values is in line with those reported for the general adult population [34–38], also for the elderly population [39, 40]. After stratifying the sample by age and gender, missing values were mostly below 3% across all five dimensions and, hence, were lower than previously published rates for the elderly population [41–44]. Further, we observed slightly increasing rates of missing responses to the descriptive system with higher age, which was already described elsewhere. However, this gradient was not as pronounced as in other studies [10, 40, 47, 48]. At the dimension level, self-care and anxiety/depression had somewhat higher proportions of missing values. While there is evidence that anxiety/depression may cause response issues and some embarrassment in the elderly population [49, 50], a higher prevalence of missing values in self-care was instead seen in patient samples with dementia [45, 46]. Furthermore, gender did not seem to be associated with completeness of the descriptive system, since men and women had similar frequencies of missing values. Nonetheless, evidence is inconclusive on the role of gender regarding this, as other studies suggest [35, 47]. With respect to the EQ VAS, we found slightly more absolute missing values in comparison to the descriptive system and a steeper increase in proportions of missing values with higher age. This is not surprising given that comprehension problems with the EQ VAS are well documented in older adults [42, 49, 51]. Nevertheless, the number of missing values described in this study is at the lower end of the range that is commonly reported in older samples [10].

To the best of our knowledge, this is the first study to conduct a multi-country comparison of missing values on the EQ-5D. With respect to missing values on the five dimensions and the EQ VAS, we found some degree of variability at the country level. Variability is even more pronounced with respect to missing EQ VAS values. However, we do not have an explanation for this. Although interviewer effects appear to be an obvious explanation [52], they are implausible in view of the highly standardized procedure in SHARE and due to the paper–pencil format. (The panellist answers the questionnaire following the interview). Since cultural measurement equivalence for different language versions of the EQ-5D has been proven several times [53–56], this explanation seems unlikely as well. Nevertheless, all country-level results are within an acceptable range, which are within the limits of what was to be expected from published literature [10].

Construct validity, i.e. convergent validity and divergent validity, of the five EQ-5D dimensions and EQ VAS was sustained by confirmation of the anticipated relationships with measures of physical, mental, cognitive, and general health as well as measures of self-perceived health status. Reassuringly, we found strong convergent validity with core aspects of the EQ-5D such as physical health and generic aspects of self-perceived health status (e.g. CASP, number of symptoms, and self-perceived health), which indicates that the EQ-5D measures overlapping constructs with the aforementioned scales. This is broadly in line with earlier studies [38, 57–59]. On the other hand, there is clear evidence of divergence between the EQ-5D components and constructs measuring aspects of mental and cognitive health as well as BMI or grip strength. Due to the rather low correlation, it appears that these constructs are not well reflected by the EQ-5D. These findings support the need for additional measures supplementing the self-reported health assessment in older respondents, as cognition is known to deteriorate with higher age [60] or where hand grip strength is assessed to detect older adults at risk of physical decline [61]. The prevalence of cognitive impairment (CI) increases with age [62, 63], and CI affects the live of affected individuals, negatively impacting the autonomy [64]. Since the findings of this study show an increasing impact of physical and cognitive aspects on self-perceived health status with age, and cognitive aspects are not captured by the EQ-5D, studies investigating older persons should consider the additional use of the cognition bolt-on [65–67]. However, in general, a general measure such as the EQ-5D appears to better capture the overall impact on health of the ageing population than a measure that addresses only a single aspect or dimension of health.

Both the EQ-5D descriptive system and the EQ VAS differentiate well between groups with known health differences, thus, demonstrating evidence to support known-groups validity for the EQ-5D. For sociodemographic variables, our findings are well aligned with results from the published literature and point in the expected direction with those being female, with lower education, living alone, and being unemployed had lower health in terms of EQ VAS and more problems reported on the descriptive system [40, 57, 59, 68–72]. Similarly, we found evidence to support differences between age groups, where health decreases monotonically with increasing age with a greater dip for the oldest age group, which supports findings from earlier studies [57, 68, 71, 72]. Interestingly, four studies reported some inconsistency with regard to age and the proportion of reported problems in anxiety/depression, which seemed to plateau or even decrease in older age groups [40, 59, 71, 72]. However, this pattern was not replicated in our findings indicating known-groups validity of the anxiety/depression dimension regarding different age groups. Moreover, known-group differences based on physical and general health measures were also confirmed for the descriptive system and the EQ VAS by our findings and are in line with the hypotheses suggested by the literature [45, 71]. Additionally, this study adds new validity evidence to the literature, since both components, the descriptive system and the EQ VAS, satisfactorily differentiated among groups with different stages of cognitive health in this sample of older Europeans, whereas the literature is otherwise scarce. There is mixed published evidence with respect to the EQ VAS’s ability to detect differences among severity levels in patients with dementia [46, 73]. For the descriptive system, potential ceiling effects were described for dementia patients [74–76], where the lack of sensitivity essentially prevents satisfactory discrimination of subgroups; the only exception was anxiety/depression, which showed positive association with a severity classification measure of dementia [46]. Despite these contradictory findings from the literature, the EQ-5D was able to demonstrate different levels of problems for groups with varying cognitive health.

This study’s major strength is the large sample size available for the analysis of EQ-5D data, which provides two distinct advantages. First, the sample includes a wide spread of reported health problems and, hence, offers the potential to analyse measurement properties across the whole severity range. Second, the sample size is sufficient to detect known-group differences, whereas other studies often lack the power to detect statistically significant differences. However, some limitations need to be considered. The underlying data may be prone to a selection bias favouring respondents who are potentially healthier and more independent as those respondents are more likely to respond to a survey. Thus, severely ill, institutionalized, and non-independent respondents may be underrepresented in this sample. Consequently, the demonstrated measurement properties may not be fully representative for those respondents. Similarly, we have no information as to whether respondents required or received help when answering the EQ-5D. Hence, we cannot control for this factor, and it should be considered that feasibility aspects may be overestimated.

Moreover, there is a newer version of the EQ-5D with an extended response scale (five levels) and improved measurement properties (e.g. in terms of sensitivity and distribution properties) available [77], and based on the demonstrated superior ability of the generic EQ-5D to capture the overall impact on health of the ageing population, a shift to the refined five-level EQ-5D may be considered to provide an even more accurate assessment of health in the elderly SHARE population.

## Conclusion

In conclusion, the EQ-5D descriptive system and the EQ VAS demonstrated very good feasibility properties, construct validity, and known-groups validity in a sample of pan-European older adults. Our results provide further evidence to strengthen the use of self-administered EQ-5D in older populations achieving a high degree of instrument completion. A generic measure such as the EQ-5D seems to better capture the overall impact on health of the ageing population when compared to measures dedicated to a single aspect or dimension of health only.
